# Public attitudes towards mental health stigma in Moldova: A comparative study

**DOI:** 10.1192/j.eurpsy.2025.279

**Published:** 2025-08-26

**Authors:** J. Chihai, V. Condrat

**Affiliations:** 1 Mental health, medical psychology and psychotherapy, State Medical and Pharmaceutical University Nicolae Testemitanu; 2 IP Trimbos Institute Moldova, Chisinau, Moldova, Republic of

## Abstract

**Introduction:**

Mental health stigma remains a significant challenge in Moldova despite ongoing progress in mental health services. The negative perception and stereotypes about mental disorders have been socially ingrained, resulting in negative attitudes, fear, or even humor directed towards affected individuals. Addressing this stigma is essential to improve mental health outcomes and integrate services effectively.

**Objectives:**

The study aimed to evaluate public perceptions and attitudes towards mental health stigma in Moldova, identify common stereotypes, and suggest actions to reduce stigma.

**Methods:**

The research employed a comparative study design using a structured questionnaire across two different time points: 2018 and 2022. Data were collected through face-to-face interviews utilizing the Computer-Assisted Personal Interviewing (CAPI) method, ensuring data accuracy and consistency. The study covered eight districts, with a total sample of 2973 participants stratified by geographic region and demographic characteristics.

**Results:**

The findings highlighted that the level of acceptance towards individuals with mental health issues was moderate, with participants showing more acceptance at a distance, indicating persistent stigma. Notable differences between urban and rural areas were observed, with urban areas showing a higher level of openness. Furthermore, stigma was significantly influenced by the level of education and age of the respondents.

**Conclusions:**

Mental health services are currently available across all districts of Moldova, but there remains a need for ongoing improvements to ensure equal access to high-quality care and to minimize both geographical and stigma-related barriers. Ongoing training programs for healthcare professionals are vital for effectively reducing stigma and improving the quality of care for those facing mental health challenges, with a specific focus on non-discriminatory practices and empathetic approaches. To achieve successful mental health care, a collaborative approach that involves healthcare professionals, policymakers, and community stakeholders is essential. This multidisciplinary coordination is key to establishing a supportive and comprehensive care environment. Additionally, public awareness campaigns and educational initiatives targeting both the general population and healthcare providers are critical in reducing stigma and changing attitudes towards mental health, thereby encouraging more people to seek care. Finally, integrating mental health services into primary healthcare should be prioritized to enhance accessibility and continuity of care. This integration will also facilitate early diagnosis and timely intervention, leading to improved outcomes for patients.

**Disclosure of Interest:**

None Declared

